# An Unusual Presentation of Plantar Psoriasis: A Case Report

**DOI:** 10.7759/cureus.74864

**Published:** 2024-11-30

**Authors:** Marta N Baptista, Pedro D Cardoso, Sara D Sousa, Ricardo Ribeiro

**Affiliations:** 1 Family Medicine, Unidade de Saúde Familiar, Unidade Local de Saúde de Braga, Braga, PRT

**Keywords:** chronic dermatological conditions, multidisciplinary health care, plantar psoriasis, psoriasis diagnosis, topical corticosteroids

## Abstract

Plantar psoriasis is a chronic inflammatory skin disorder, typically characterized by erythematous plaques with thick silvery scales localized on the soles. This condition can significantly impair patients' quality of life, particularly through pain and mobility challenges. It is considered a subtype of plaque psoriasis but presents unique diagnostic and therapeutic challenges due to its specific location. We report the case of a 44-year-old female with bilateral burning and scaling of the feet, initially misdiagnosed and treated for fungal infections without success. Despite persistent symptoms, a dermatological consultation was delayed for three years. Upon reassessment, she presented with scaly lesions on the elbows and feet. Treatment with topical betamethasone and calcipotriol led to partial resolution of plantar lesions but not the elbow lesions. Following a comprehensive evaluation, the patient was diagnosed with plantar psoriasis and maintained on topical therapy, avoiding systemic treatments. Her management highlights the importance of accurate diagnosis and timely specialist referral.

This case illustrates the challenges in diagnosing plantar psoriasis, particularly when overlapping with other dermatological conditions. A conservative treatment approach with topical steroids proved effective in managing symptoms. The patient’s delayed referral underscores the need for prompt specialist care to mitigate prolonged discomfort. Additionally, comorbidities like anxiety and depression may exacerbate the patient's symptoms, emphasizing the importance of integrated care that addresses both physical and psychological aspects. This case emphasizes the necessity of timely and accurate diagnosis, early referral for specialist care, and a multidisciplinary management approach for chronic dermatological conditions such as plantar psoriasis. Further research is warranted to explore the interplay between psychological factors and dermatological conditions to improve patient outcomes.

## Introduction

Plantar psoriasis is a chronic inflammatory skin disorder that specifically affects the soles of the feet. It is characterized by well-demarcated, erythematous plaques with thick silvery scales. It can significantly impair a patient's quality of life not only because of the aesthetic aspect of the disease but also due to pain and difficulty in walking causing limitation in mobility [1,2].

Plantar psoriasis is a subtype of plaque psoriasis and shares many of its pathophysiological mechanisms, but the unique localization on the soles poses specific challenges in diagnosis, management, and treatment. Psoriasis is a dermatological condition affecting approximately 2-3% of the global population [3]. Palmoplantar psoriasis, which affects the palms of the hands and soles of the feet, is a less common manifestation, occurring in about 3-4% of patients with psoriasis [3]. The specific prevalence of plantar psoriasis is not well-documented separately from palmoplantar psoriasis, but it affects both genders equally and can occur at any age, with a peak onset in adults [4].

The diagnosis of plantar psoriasis is primarily clinical, relying on the characteristic appearance of lesions and patient history, so it is important to check the palmar hands for skin lesions and ask the patient if there is a history of psoriasis or any other skin rashes on the body [1,2].

Dermoscopy can be a valuable diagnostic tool, as it helps differentiate psoriasis from other similar conditions by revealing features such as uniform vascular patterns and silvery-white scales [5]. In uncertain cases, a skin biopsy may be warranted, which typically shows histopathological features like hyperkeratosis, parakeratosis, elongation of rete ridges, and a perivascular lymphocytic infiltrate [6].

## Case presentation

A 44-year-old female was presented with a medical history of anxiety and depression and was a current smoker. Family history revealed unspecified dermatological conditions affecting her father and sister. She was taking sertraline at a dosage of 100 mg daily and reported no known drug allergies.

The patient presented to her primary care physician in February 2021 with a chief complaint of bilateral burning sensations and scaling of the feet, which she described as causing significant discomfort in her daily life.

Approximately six months prior, she had removed "dead skin" from her heels, after which a scaly lesion persisted. At that time, on her own initiative, she was evaluated by a dermatologist who prescribed topical and oral antifungal medications and performed a skin biopsy. However, these treatments did not improve her symptoms, and the skin biopsy was negative for fungal infection.

**Figure 1:**
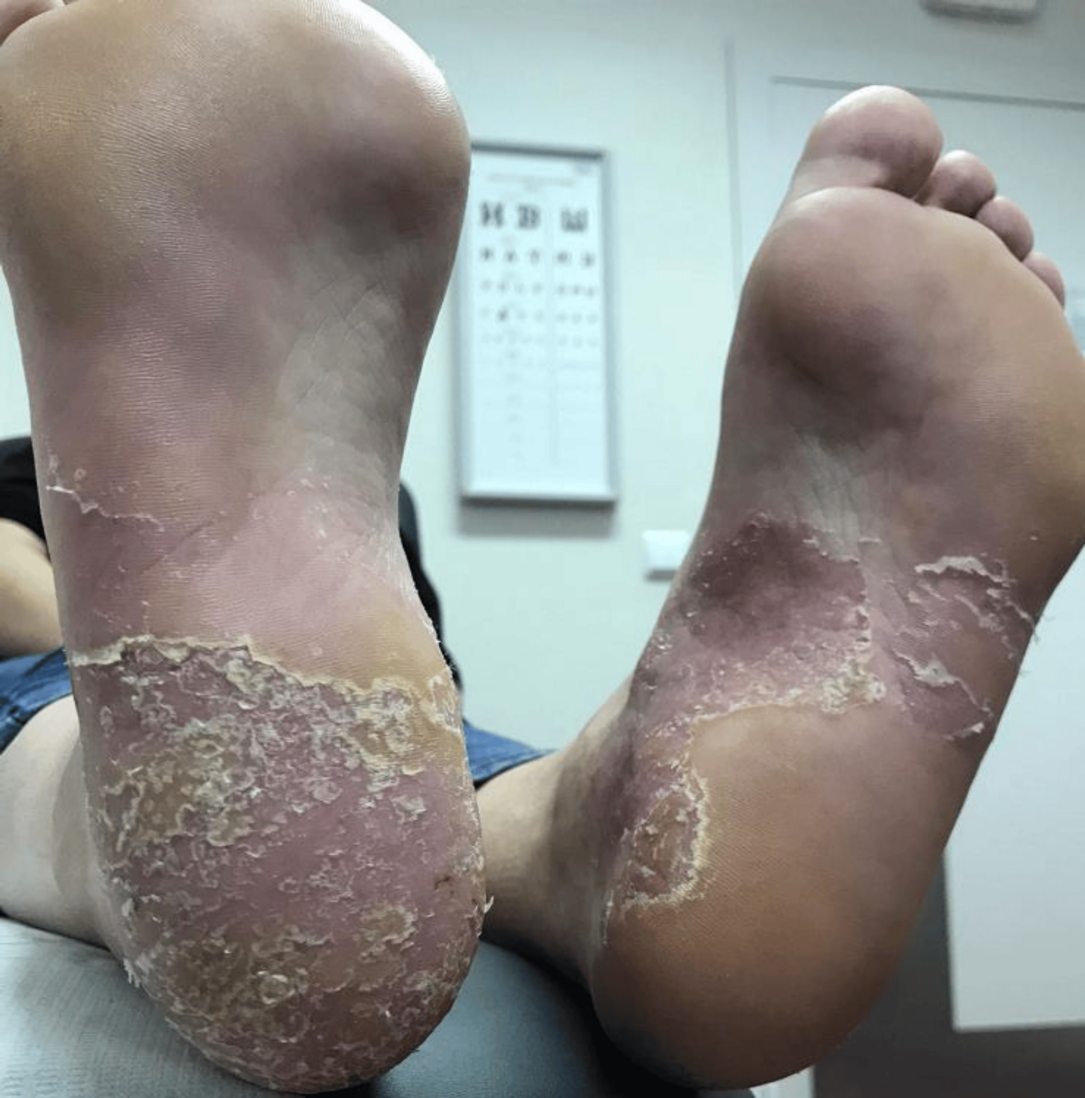
Condition of the patient's heels before treatment Signs of scaling and hyperkeratosis on the bilateral heels, accompanied by areas of erythema.

At the time of this visit, there were signs of scaling and hyperkeratosis on the bilateral heels, accompanied by areas of erythema (see Figure 1).

**Figure 2:**
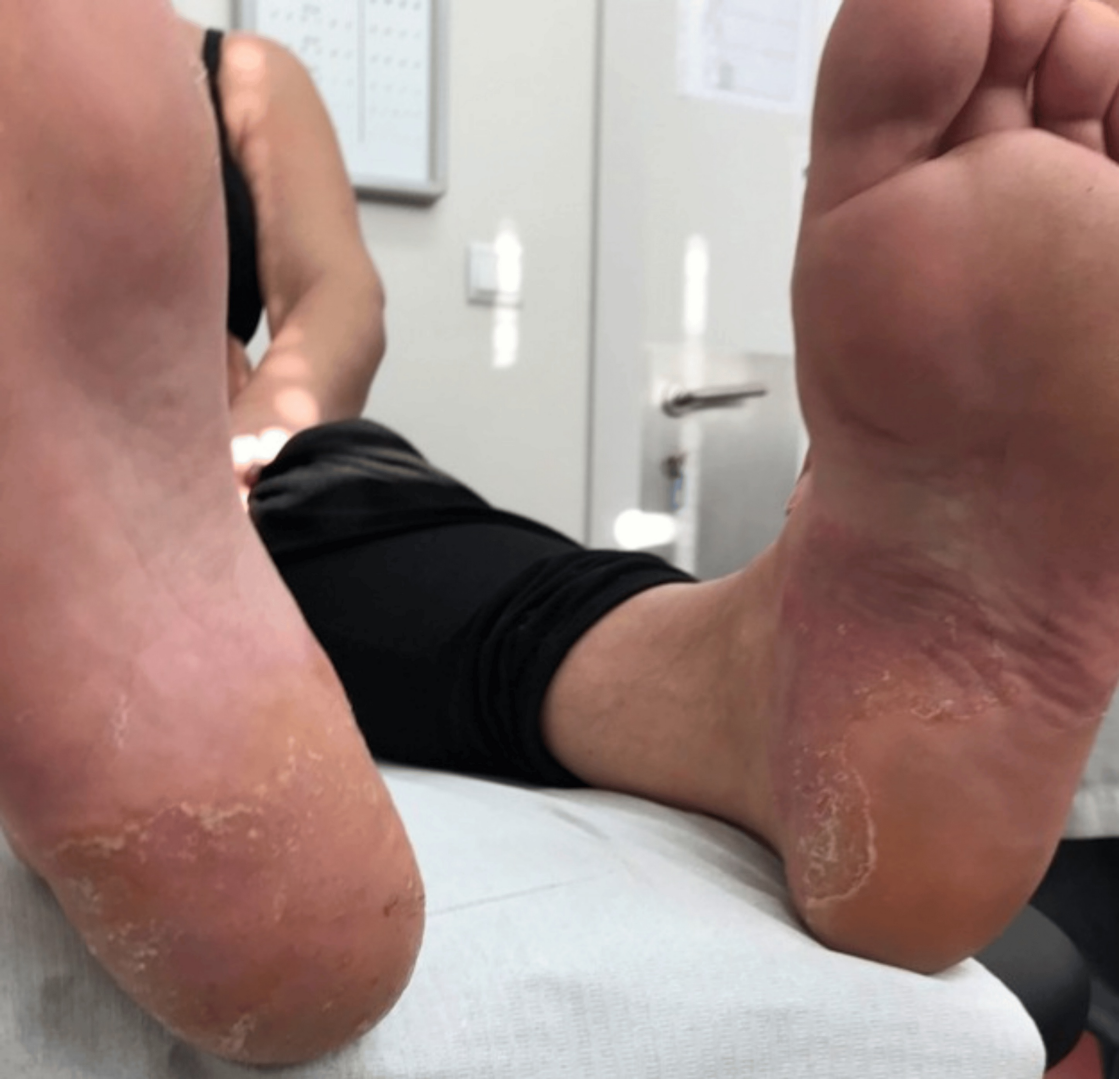
Condition upon four weeks treatment Notable improvement in the lesions on the patient's heels.

The differential diagnoses included hypersensitivity or irritation reactions, fungal or bacterial infections, and plantar psoriasis. The patient was referred to a dermatologist for a comprehensive evaluation of her plantar and palmar surfaces; however, this appointment was delayed by three years. During her second consultation with her primary care physician in March 2021, the patient reported that her symptoms had persisted and noted the recent emergence of well-defined scaly lesions on both elbows. Given the etiology of her chronic discomfort, she was initiated on urea 30% exfoliating cream, bacitracin plus retinol, and a combination of betamethasone and calcipotriol. Upon reevaluation four weeks later, there was a notable improvement in the lesions on her feet (see Figure 2); however, the lesions on her elbows remained unchanged but were not bothersome. The patient was instructed to discontinue treatment, but within two weeks, erythema and burning sensations recurred. Resuming the topical steroid regimen led to the resolution of the lesions once again.

In April 2024, the patient underwent further evaluation by a dermatologist, who diagnosed her with plantar psoriasis and prescribed betamethasone combined with calcipotriol for on-demand use. The dermatologist opted against biologic therapy, reasoning that the current topical treatments appeared to be effective and represented a more conservative management approach.

## Discussion

This case highlights the complexities inherent in diagnosing and managing chronic dermatological conditions, specifically plantar psoriasis, in the context of overlapping clinical features and prior treatment attempts. The patient, a 44-year-old female with a history of anxiety and depression, presented with persistent symptoms of burning and scaling feet, which significantly impacted her quality of life. Importantly, her prior treatments with antifungal therapies proved ineffective, and subsequent diagnostic evaluations, including a skin biopsy, were negative for fungal infections.

The pathogenesis of plantar psoriasis involves a complex interplay of genetic, environmental, and immunological factors [5]. Environmental triggers, such as trauma (Koebner phenomenon), infections, and psychological stress, can exacerbate the condition [7].

This skin disorder may be initiated or exacerbated by some drug intake. Some of the most common medications known to trigger or worsen existing psoriasis include lithium, gold salts, beta-blockers, and antimalarials. There is also a clinical report on olmesartan [8]. The pathophysiology is yet to be discovered.

Plantar psoriasis can be challenging to differentiate from other conditions affecting the soles of the feet, particularly fungal infections, bacterial infections, and hypersensitivity reactions. Tinea pedis presents with erythematous, scaling lesions, often in a moccasin distribution, but lacks the silvery scales and well-demarcated plaques seen in psoriasis. Bacterial infections, such as impetigo or erysipelas, may cause erythema and swelling, often with pain and warmth, which are not characteristic of psoriasis. Hypersensitivity reactions, including contact dermatitis or drug eruptions, typically cause acute erythema and inflammation, often linked to a specific trigger, and are distinguished by the absence of silvery scales and the presence of vesicles or blisters, with patch testing or biopsy providing further clarity. By recognizing these differences and utilizing appropriate diagnostic tests, clinicians can accurately diagnose and treat the underlying condition, ensuring better outcomes for patients [5,6].

As evidenced by the treatment course initiated by the dermatologist, the therapeutic approach was initially conservative, focusing on topical agents that have demonstrated efficacy in managing psoriasis [9]. Betamethasone and calcipotriol, as a combination therapy, are well-documented for their effectiveness in reducing inflammation and scaling associated with psoriasis while minimizing systemic side effects compared to systemic therapies [10].

In terms of topical therapies, potent topical corticosteroids, retinoids and vitamin D, salicylic acid, and emollient analogues are considered first-line treatments. These are generally safer and more cost-efficient for long-term use, making it an excellent first-line treatment option [9]. In 80% of patients diagnosed with plantar psoriasis, topical steroids are the primary treatment option because of their anti-inflammatory, immunosuppressive, and antiproliferative properties [11]. These agents reduce inflammation and hyperproliferation of keratinocytes. Other topical treatments, such as calcineurin inhibitors and keratolytic agents, can also be beneficial, particularly for maintenance therapy [9].

The delayed referral for further dermatological evaluation, spanning three years, underscores the need for timely consultations in managing chronic skin conditions. Delays in specialty care can lead to prolonged symptoms and increased patient discomfort, as seen in this case. In primary care settings, access to dermoscopy and biopsy is often limited, necessitating a clinical diagnosis that relies on the expertise of a qualified specialist. Current literature emphasizes the importance of early and accurate diagnosis, as well as timely access to specialist care, to achieve optimal management of psoriasis and other chronic dermatological disorders [10].

Moreover, the patient's history of anxiety and depression can complicate her clinical presentation and treatment adherence. These comorbidities are known to exacerbate the perception of pain and discomfort associated with dermatological conditions [12]. Therefore, an integrated management approach that addresses both the dermatological and psychological aspects is crucial for enhancing patient outcomes.

## Conclusions

In conclusion, this case exemplifies the need for a comprehensive understanding of chronic dermatological conditions and highlights the role of multidisciplinary care in optimizing patient management. The patient’s experience underscores the significance of effective diagnosis and appropriate therapeutic interventions, especially in the context of limited primary care resources.

The prolonged delay in specialist referral further emphasizes the critical need for timely access to dermatological care, which is essential for mitigating chronic symptoms and improving patient quality of life. Additionally, recognizing and addressing comorbid psychological conditions is vital, as they can contribute to both symptom severity and treatment adherence. Further research is warranted to investigate the impact of psychological factors on the presentation and treatment of skin disorders and to refine strategies for enhancing patient access to dermatological care.
